# New data on the stem and leaf anatomy of two conifers from the Lower Cretaceous of the Araripe Basin, northeastern Brazil, and their taxonomic and paleoecological implications

**DOI:** 10.1371/journal.pone.0173090

**Published:** 2017-03-03

**Authors:** Maria Edenilce Peixoto Batista, Delmira da Costa Silva, Marcos A. F. Sales, Artur A. Sá, Antônio A. F. Saraiva, Maria Iracema Bezerra Loiola

**Affiliations:** 1 Programa de Pós-Graduação em Ecologia e Recursos Naturais, Departamento de Biologia, Universidade Federal do Ceará, Fortaleza, Ceará, Brazil; 2 Departamento de Ciências Biológicas, Universidade Estadual de Santa Cruz, Santa Cruz, Bahia, Brazil; 3 Programa de Pós-Graduação em Geociências, Instituto de Geociências, Universidade Federal do Rio Grande do Sul, Porto Alegre, Rio Grande do Sul, Brazil; 4 Departamento de Geologia, Universidade de Trás-os-Montes e Alto Douro, Vila Real, Portugal; 5 Centro de Geociências, Universidade de Coimbra, Coimbra, Portugal; 6 Departamento de Ciências Biológicas, Universidade Regional do Cariri, Crato, Ceará, Brazil; 7 Departamento de Biologia, Universidade Federal do Ceará, Fortaleza, Ceará, Brazil; Institute of Botany, CHINA

## Abstract

*Pseudofrenelopsis* and *Brachyphyllum* are two conifers that were part of the Lower Cretaceous (Aptian) taphoflora of the Crato Formation, Araripe Basin, northeastern Brazil. The former genus includes, so far, *P*. *capillata* and indeterminate species, whilst the latter is mainly represented by *B*. *obesum*, the most common plant megafossil recovered from that stratigraphic unit. Here, the stem and leaf anatomy of *Pseudofrenelopsis* sp. and *B*. *obesum* specimens is revisited, including the first report of some epidermal and vascular traits for both taxa from the Crato Formation. Along with its paleoecological significance, the new data suggest the presence of more than one *Pseudofrenelopsis* species in the Aptian taphoflora of the Araripe Basin and further support the taxonomic placement of *B*. *obesum* within Araucariaceae.

## Introduction

During most of the Mesozoic, landscapes were dominated worldwide by gymnosperms. Throughout the Upper Cretaceous, the gymnosperm-dominated floras became restricted mainly to high latitudes as they were gradually replaced by angiosperms, whose evolutionary radiation had begun in low latitudes during the Lower Cretaceous and then extended globally [1–4].

*Pseudofrenolopsis* and *Brachyphyllum* are two conifers among the most abundant and diverse gymnosperms of the Mesozoic with their demise at the end of the Cretaceous [5–8]. The first genus is placed in Cheirolepidiaceae, a family commonly regarded as xeromorphic due to its morphology and anatomy adapted to arid climates [9,10]. However, the second one was already attributed to different families, like Araucariaceae, Cheirolepidiaceae, Cupressaceae, and Podocarpaceae, making difficult the taxonomic placement of the about fifty species of this genus [8]. For instance, some *Brachyphyllum* species, like *B*. *mamillare* Lindley and Hutton, were classified as Araucariaceae, based on leaf and cuticle features [11,12]. Whereas, other species, like *B*. *crucis* Kendal, *B*. *patens* (Miquel) and *B*. *ardenicum* Harris, show affinity with Cheirolepidiaceae [6,13,14].

The aforementioned genera are found together both in the Aptian Crato and the Albian Romualdo Formations of the Santana Group (Araripe Basin, northeastern Brazil), represented by *P*. *capillata* Sucerquia, Bernardes-de-Oliveira et Mohr, *B*. *castilhoi* Duarte and *B*. *obesum* Heer, along with indeterminate species [15–17]. Particularly, the latter species is the most common taxon among the plant remains from both formations. It was originally described from the Aptian of the Almargem Basin, Portugal, with further records from different places of the world, like the United Kingdom, China, and Japan [17–20]. Morphological evidence suggests *B*. *obesum* either as a member of Araucariaceae or Cheirolepidiaceae [21–23], a matter of controversy between authors regarding the taxonomic diversity of the Araripe Basin. In this sense, Kunzmann et al. [21] placed *B*. *obesum* in Araucariaceae, describing anatomical traits that supported their taxonomic proposal. On the other hand, Sucerquia [22] supported the attribution of *B*. *obesum* to Cheirolepidiaceae based on the local abundance of *Classopollis*-type pollen. Nevertheless, these authors also mentioned the necessity of more evidence in order to further corroborate their inference.

Anatomical features of vegetative plant organs can be added to the external morphological traits, helping to solve taxonomic disputes [24–26]. For instance, vascular structures, like growth rings, tracheids, and intercellular canals, and epidermal features are often used for that purposes (e.g., [25–27]). However, probably due to aspects related to the preparation of samples and the obtainment of good results, transverse sections, for example, are less often used in paleobotanical studies with fragile remains, limiting the recovered amount of data on some important features [28]. In fact, this technique is more common in studies with large permineralized trunks (e.g., [29,30]).

In this work, we present additional data on the anatomy of *B*. *obesum* and *Pseudofrenelopsis* sp. branches from the Crato Formation of the Araripe Basin based on new specimens. Although focusing mainly on vascular traits, our study also comprises epidermal structures. The new information contributes to the knowledge on the anatomy of these conifers and supports the presence of more than one *Pseudofrenelopsis* taxa in the Araripe Basin and corroborates the taxonomic placement of *B*. *obesum* in Araucariaceae.

## Geological setting

**Fig 1:**
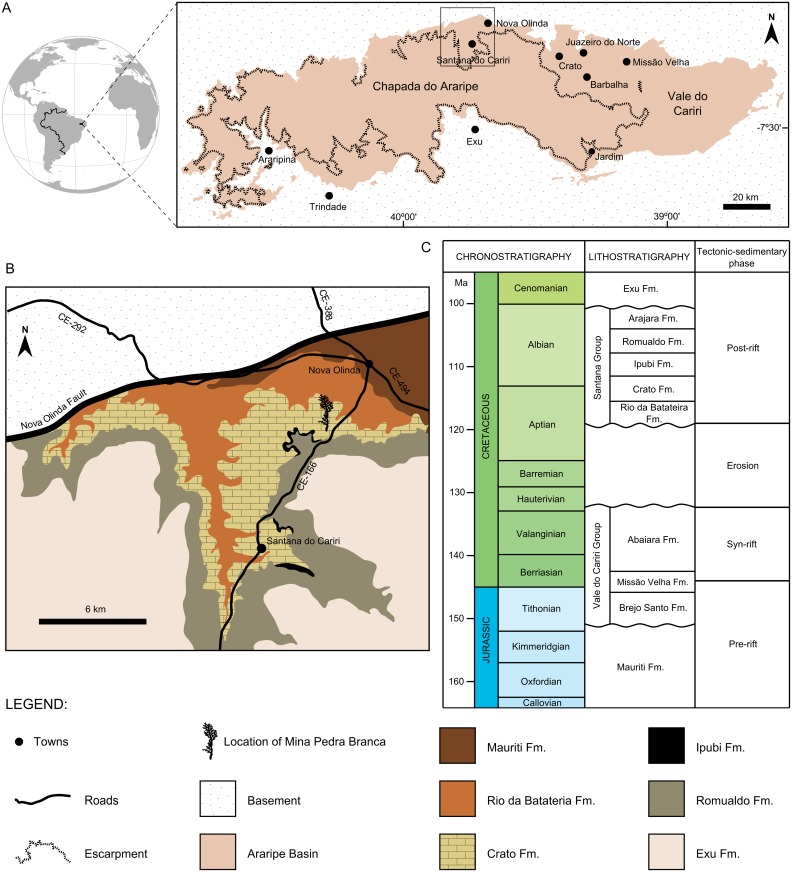
Geology and stratigraphy of the Araripe Basin, northeastern Brazil. (A) Geographical location of the Araripe Basin and its main geomorphological features. (B) Simplified geological map of the area indicated by the rectangle in (A). (C) Stratigraphic scheme of the Araripe Basin. (A) is modified from Alroy [36] and Assine [35], whereas (B) and (C) are based on Matill [37] and Valença et al. [32], respectively.

The Araripe Basin is located in the central part of the Borborema Tectonic Province and extends throughout the Brazilian states of southern Ceará, northwestern Pernambuco, and eastern Piauí (Fig 1). It is the most extensive intracratonic basin of northeastern Brazil and covers an area of about 9,000 km^2^ [31,32]. Geomorphologically, its main features are a plateau and a valley called as Chapada do Araripe and Vale do Cariri, respectively, where crops out a rich fossiliferous deposit known as the Santana Group [32,33]. This group is part of the post-rift sequence comprising the Rio da Batateira, Crato, Ipubi, Romualdo, and Arajara formations and is regarded as Aptian–Albian in age [32–34] (but for a different stratigraphic proposal see, for instance, [31,35]).

Particularly important for this study, the Crato Formation consists of finely laminated limestones, locally displaying gentle undulations with wave truncations and indicating a lacustrine carbonate depositional environment, typical of calm waters with episodic high-energy events [32]. This formation has yielded many fossil specimens, including invertebrates, vertebrates, plants, trace fossils, and palynomorphs, which together indicate a probable upper Aptian age for this unit [38]. Fishes are abundant, especially the occurrence of the genus *Dastilbe*, generally preserved by pyritization, limonitization or carbonization processes [38,39]. In general, the taphoflora of the Crato Formation is quite diverse, being represented by sphenophytes, lycophytes, monilophytes, gnetaleans, conifers, and angiosperms [2,40,41]. Among the conifers are represented the families Cheirolepidiaceae and Araucariaceae, both characterized by large trees [2,41,42].

## Materials and methods

The studied material comprises specimens properly housed at the following public institutions: Laboratório de Paleontologia of the Universidade Regional do Cariri (LPU), Crato Municipality, and Museu de Paleontologia de Santana do Cariri (MPSC), Santana do Cariri Municipality, both located in the state of Ceará, Brazil. It includes a stem of *Pseudofrenelopsis* sp. (LPU 312 PL) and fragments of leaves and stems of *Brachyphyllum obesum* (LPU 242 PL, MPSC PL 551, 580, and 802) from the Crato Formation of the Araripe Basin. All the specimens were collected at Mina Pedra Branca, a limestone quarry located between Santana do Cariri and Nova Olinda (Fig 1B).

For sectioning, were selected the best three-dimensionally preserved fragments. As the material is, in general, brittle and can easily be fractionated, an epoxy resin (RQ-01 00/RF, Alpha Resiqualy^®^) was used for cold impregnation of the fragments, which were, then, kept for about 48 hours at room temperature. The slices were prepared only after the impregnation process.

The analysis of the anatomy of the stem fragments in radial longitudinal and transverse sections were performed with the HITACHI^®^ Scanning Electron Microscope, Model TM3000, at the Laboratório de Microscopia Eletrônica of the Universidade Federal do Ceará, Fortaleza Municipality, Brazil. In order to investigate the tracheid and cross-field pits and the stomatal arrangement, the remaining material was processed and analyzed both at the Central Analítica of the Universidade Federal do Ceará and Centro de Microscopia Eletrônica of the Universidade Estadual de Santa Cruz (UESC), Ilhéus Municipality, Brazil. At the former institution, remains were attached to stubs with carbon tape and covered with a 20-nm thick gold layer and, then, analyzed under the Scanning Electron Microscope INSPECT 50, FEI Company. At the latter, samples of fossil fragments were attached to stubs with carbon tape and covered with a 30-nm thick gold layer and, later, observed under the Scanning Electron Microscope Quanta 250kV, FEI Company.

## Results

### Systematic paleontology

Cheirolepidiaceae Takhtajan

*Pseudofrenelopsis* (Nathorst) emend. Watson

*Pseudofrenelopsis* sp.

**Fig 2:**
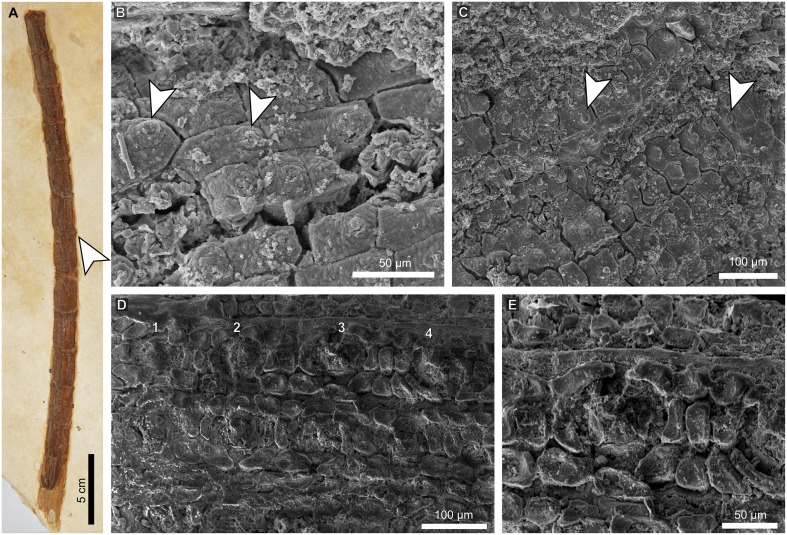
Epidermal features of a *Pseudofrenelopsis* sp. stem from the Crato Formation, Araripe Basin, northeastern Brazil. (A) Specimen LPU 312 PL. (B and C) Papillate epidermal cells from the internode basal region. (D) Stomatal rows from the internode middle region. (E) Detail of a stellate stomatal pit and ordinary epidermal cells without papillae. Arrows point to the insertion point of one leaf at the node (A) and papillae (B and C). Numbers refer to four stomatal apparatus in a single row.

**Fig 3:**
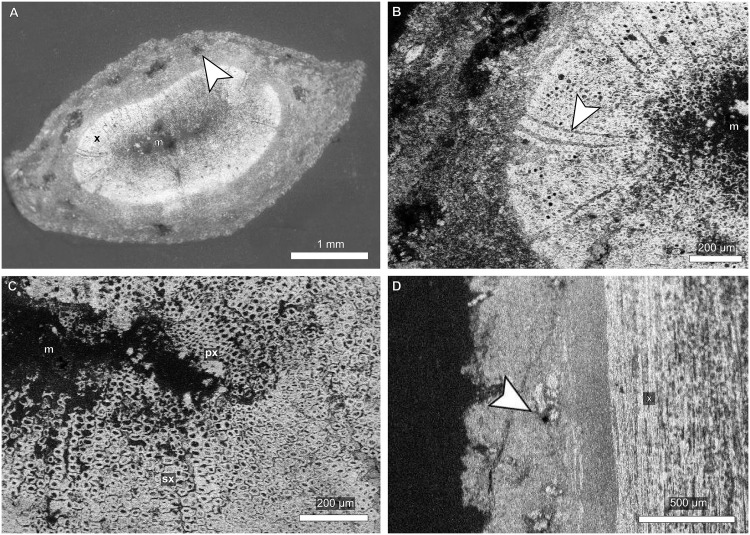
Vascular features of a *Pseudofrenelopsis* sp. stem from the Crato Formation of the Araripe Basin, northeastern Brazil. (A) Transverse section of the stem. (B) Close view of the xylem region. (C) Close view of the medullary region, from which tracheid diameters decrease gradually in the secondary xylem. (D) Longitudinal section of the stem. Arrows point to secretory canals (A and D) and a parenchyma ray (B). The abbreviations “m”, “x” “px”, and “sx” refer to the medullary, xylem, primary xylem and secondary xylem regions, respectively.

**Fig 4:**
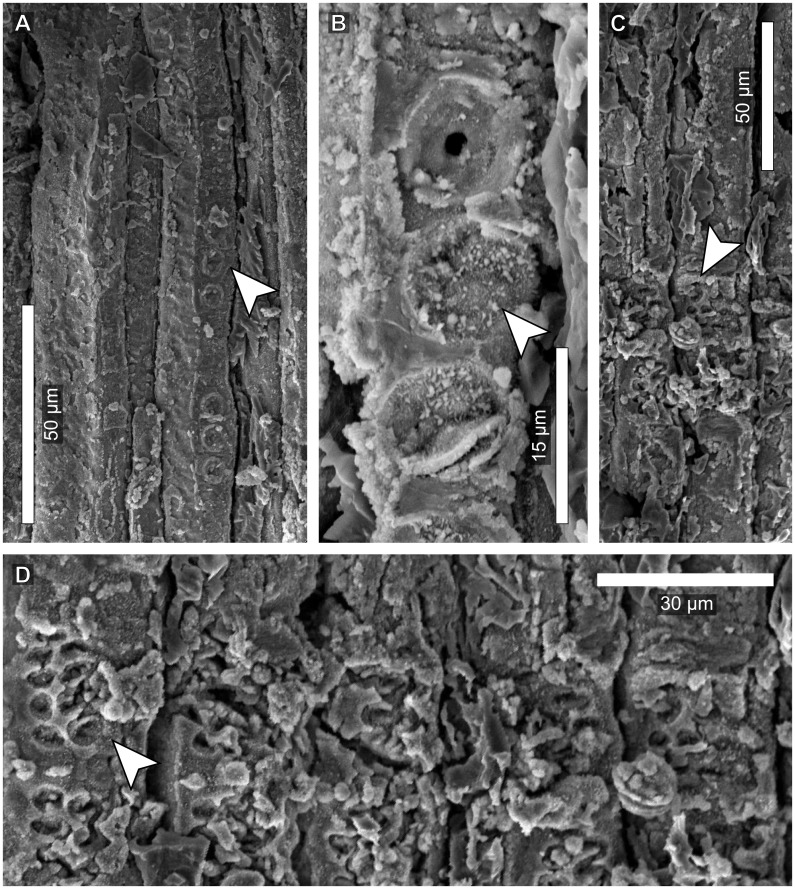
Vascular features of a *Pseudofrenelopsis* sp. stem from the Crato Formation of the Araripe Basin, northeastern Brazil. (A) Longitudinal view of a tracheid with pits on one of its sub-radial facets. (B) Detail of an areolate pit with its preserved membrane. (C) A cross-field. (D) Detail of cross-field pits. Arrows point to tracheid pits (A), a preserved membrane (B), a cross-field (C), and a cross-field pit (D).

**Studied material.** LPU 312 PL (Figs 2–4).

### Description

Specimen LPU 312 PL is an unbranched stem fragment of *Pseudofrenelopsis* sp. measuring 6 mm in width and 120 mm in length (Fig 2A). Leaves were not preserved, but their insertion points can be seen, indicating one leaf per node in a spiral phyllotaxy, a typical feature of that genus. On average, internodes are 10 mm in length. Under SEM analyses, it was possible to observe epidermal cells arranged in longitudinal rows. Those cells at the base of the internodes are either rectangular or square in shape and bear a projection at one of their ends, which characterizes them as papillate (Fig 2B and 2C). Also, there are stomata arranged in longitudinal rows, which are intercalated with two or three rows of ordinary epidermal cells with heavily cutinized anticlinal walls (Fig 2D). In each stomatal row, stomatal apparatus are often separated by two or three ordinary epidermal cells. Among these, those close to the stomata do not bear papillae. Classified as actinocytic, stomatal apparatus are rounded in shape and have an average of five subsidiary cells, which likely bore papillae that, in turn, gave a stellate pattern to the stomatal pits (Fig 2E). It was not possible to determine the orientation of the stomatal pits insofar as they were covered by diagenetic materials. At the bases of the internodes, stomata are absent.

Stem samples analyzed in transverse sections showed an ample cortical region with secretory canals (Fig 3A). It is also possible to see the medullary region, the secondary xylem formed by angular (square) tracheids and narrow parenchyma rays (Fig 3B).

The presence of different growth patterns of the xylema is noticed, which succeed each other gradually. In the primary xylem, tracheids are more compact close to the medulla, with few intercellular spaces and reduced diameters (Fig 3C). However, in the secondary xylem, younger cells, close to the cambium, have reduced lumina, due to the thickening of their walls, and few intercellular spaces. In radial longitudinal view, secretory canals were seen in the cortical region (Fig 3D).

In the longitudinal section, latewood tracheids were observed with reduced intercellular space, whereas the earlywood presented more spaced cells. Some tracheids present two sub-radial facets (Fig 4A) and have round and areolate intervascular pits arranged in a single row, with preserved membranes (Fig 4B). Each cross-field presents around ten pits, which are distributed in five or six rows and have round openings (Fig 4C and 4D).

### Systematic paleontology

Araucariaceae Henkel et Hochstetter

*Brachyphyllum* Lindley et Hutton emend. Harris

*Brachyphyllum obesum* Heer sensu Duarte

**Fig 5:**
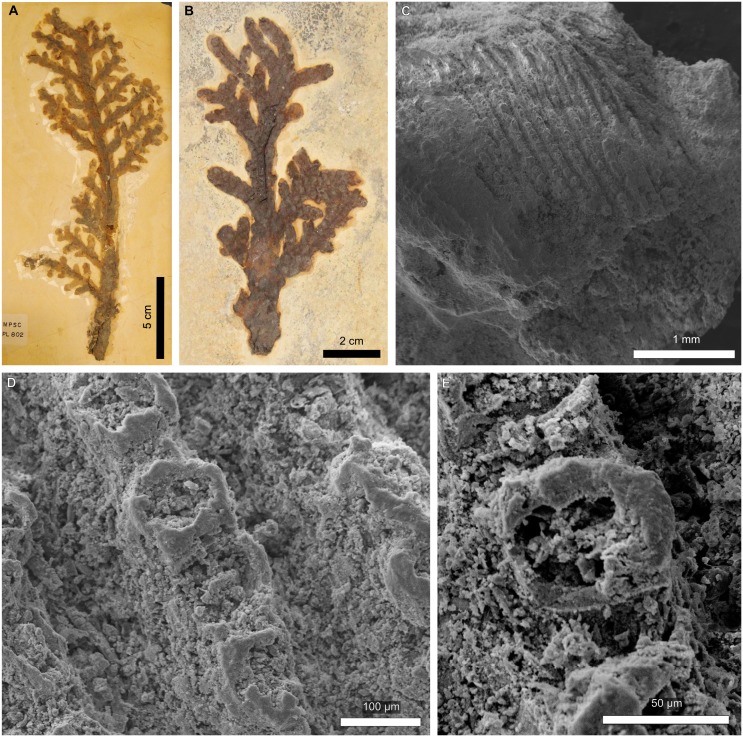
Epidermal features of *Brachyphyllum obesum* leafy branches from the Crato Formation of the Araripe Basin, northeastern Brazil. (A) Specimen MPSC PL 802. (B) Specimen LPU 242 PL. (C) Striated scale-like leaves of MPSC PL 802. (D) Stomata of MPSC PL 802 arranged in prominent longitudinal rows. (E) Detail of a stomatal apparatus of MPSC PL 802 with a possible perpendicular pit.

**Fig 6:**
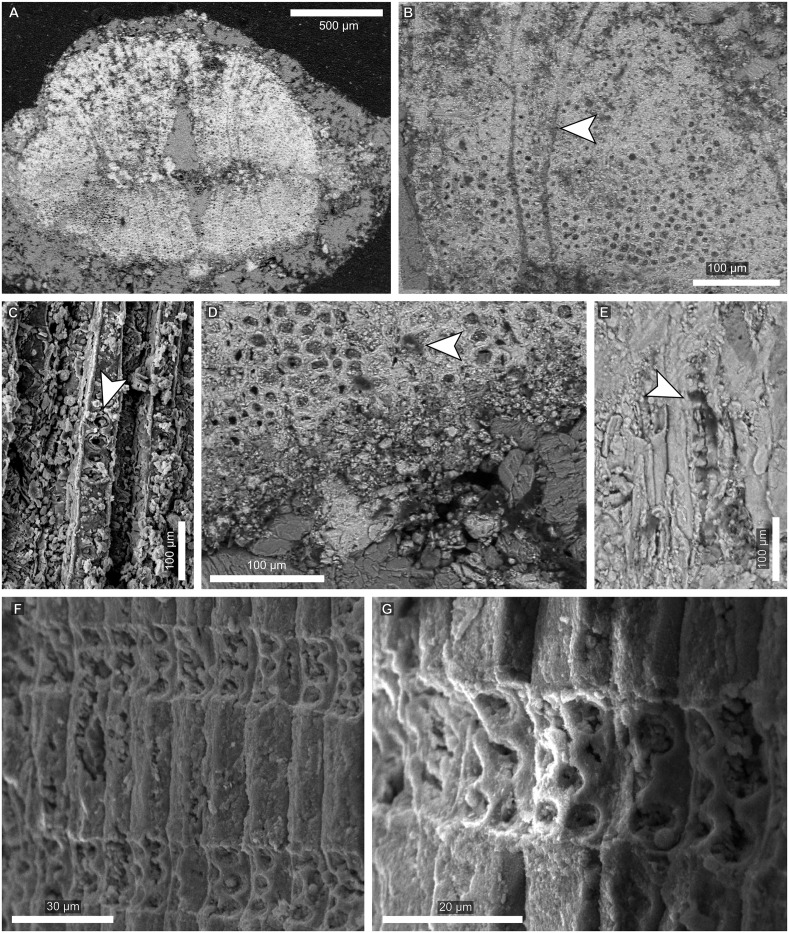
Vascular features of *Brachyphyllum obesum* branches from the Crato Formation of the Araripe Basin, northeastern Brazil. (A) Transverse section of the stem showing the cortical, xylem, and compressed medullary regions of LPU 242 PL. (B) Detail of the xylem region of LPU 242 PL in transverse view and the uniformly sized tracheids. (C) A tracheid facet of MPSC PL 580 with areolate pits. (D) Close view of the xylem region of LPU 242 PL, including resin plugs. (E) Longitudinal view of a tracheid of LPU 242 PL and its wall thickening. (F) Araucarioid cross-field pits of MPSC PL 580. (G) Detailed of the araucarioid cross-field pits of MPSC PL 580. Arrows point to a uniseriate parenchyma ray (B), a tracheid pit (C), a resin plug (D), the wall thickening of a tracheid (E), cross-fields (F), and cross-field pits (G).

**Studied material.** LPU 242 PL, MPSC PL 551, 580, and 802 (Figs 5 and 6).

### Description

*Brachyphyllum obesum* samples comprise leafy branches with alternate branching pattern and length varying between 150 and 200 mm (Fig 5A and 5B). Leaves are scale-like, rhomboid, abaxially striated, imbricate, and helically arranged, as typical of *B*. *obesum*. From the analysis of *B*. *obesum* specimens, it was possible to observe stomata on the abaxial side, which are arranged in continuous longitudinal rows (Fig 5C). Stomata are rounded to oval in shape (Fig 5D). The anticlinal walls of the subsidiary cells looked prominent, but the guard cells were not preserved. However, these were possibly sunken as in other *Brachyphyllum* specimens, including *B*. *obesum* (e.g., [13,21]). Despite the poor preservation, which did not allow the observation of ordinary epidermal cells, the stomatal apparatus suggest that their ostioles were perpendicular in relation to the stomatal rows (Fig 5D and 5E). In the stem, epidermal structures were, in general, not observable, insofar as the scale-like leaves covered them completely and internode spaces were considerably short.

In transverse sections, the cortical region was not very thick. The medullary region was compressed, which may have been due to taphonomic processes (Fig 6A). The xylem is composed by oval to rounded tracheids with uniform diameters and few or no intercellular space. Their walls were thin and their lumina, ample. There is no evidence of different growth patterns. Narrow parenchyma rays were observed and were likely uniseriate (Fig 6B). In radial longitudinal sections, tracheids also showed uniseriate and areolate pits and angular outlines (Fig 6C). There were resin plugs in the xylem (Fig 6D) and wall thickenings in the tracheids could be seen longitudinally (Fig 6E). Cross-field pits are of the araucarioid type and each cross-field presents five to eight pits distributed in three or four rows but with a tendency for crowding (Fig 6F and 6G).

## Discussion

### Significance of *Pseudofrenelopsis* anatomical features

The Brazilian record of *Pseudofrenelopsis* comes from the Lima Campos [43] and Araripe basins where only *P*. *capillata* was identified so far [16]. However, given that most *Pseudofrenelopsis* species are diagnosed by epidermal anatomical structures and studies focusing on them are relatively scarce regarding northeastern Brazilian specimens, the number of species was possibly greater.

Specimen LPU 312 PL, attributed to *Pseudofrenelopsis* sp., shared many features with other congeneric species. For instance, papillate epidermal cells are among the observed features of *P*. *capillata*, *P*. *dalatzensis*, *P*. *papillosa*, *P*. *varians*, *P*. *parceramosa*, *P*. *nathorstiana*, and *P*. *liupanshanensis* [16,28,44–48] (Table 1). However, in the middle region of the internodes of LPU 312 PL, where the concentration of stomata was higher, there was no papilla in ordinary epidermal cells, differing from the aforementioned species. Furthermore, *P*. *guixiensis*, *P*. *gansuensis*, and *P*. *intermedia* do not have papillae in the internode region [49–51].

**Table 1:** Comparison of epidermal features of known *Pseudofrenelopsis* species.

Species	Number of leaves per node	Ordinary cells of the epidermis of the internode	Number of subsidiary cells	Subsidiary cells	Shape of the stomata	References
*P*. *varians*	1	With papillae and hairs	4–9	With papillae overhanging stomatal pit	Rounded or sub-rounded,	[46]
*P*. *dalatzensis*	1	With papillae and hairs	5–6	With papillae overhanging stomatal pit	Rounded to elliptical, actinocytic, stellate	[44,45]
*P*. *papillosa*	1	With papillae	4–8	With papillae overhanging stomatal pit	Rounded	[44]
*P*. *nathorstiana*	1–2	With papillae	4–7	With papillae	Rouded, usually monocyclic	[47]
*P*. *liupanshanensis*	1	With papillae	5–6	With papillae	Rounded or sub-rounded, haplocheilic	[48]
*P*. *glabra*	1	Without papillae or hairs	6–8	With papillae overhanging stomatal pit	Rounded	[52]
*P*. *guixiensis*	1	Without papillae	4–6	With papillae	Rounded or subrounded, haplocheilic	[49]
*P*. *gansuensis*	1	Without papillae or hairs	6–9	Without papillae	Elliptical to sub-rounded, actinocytic	[50]
*P*. *heishanensis*	1	Usually without papillae or hairs	5–7	With papillae overhanging stomatal pit	Elliptical to sub-rounded, stellate	[44]
*P*. *parceramosa*	1	With papillae (variable)	4–6	With or without papillae protruded to stomatal pit	Elliptical or rounded	[28,46,53,54]
*P*. *capillata*	1	With papillae and long hairs	5–6	With papillae overhanging stomatal pit	Elliptical to rounded	[16]
*P*. *intermedia*	1	Without papillae or hairs	6–11	Without papillae	Rounded or sub-rounded, haplocheilic	[51]
LPU 312 PL	1	With papillae, except in the region where the stomata are concentrated	5	Not observed	Rounded, stellate, actinocytic	This paper

Despite the differences highlighted above, the actinocytic stomata seen in LPU 312 PL are also found in *P*. *gansuensis* and *P*. *dalatzensis* [44,45,50]. Subsidiary cells, in number of five, are similar to those of *P*. *capillata*, *P*. *dalatzensis*, and *P*. *liupanshanensis* [16,45,48]. Despite subsidiary cells being only partially preserved, it was possible to see the stellate opening formed by the contact between them. This type of opening is quite common in *Pseudofrenelopsis* species and seems to be a natural consequence of the presence of papillae in the subsidiary cells, as observed in *P*. *heishanensis*, and *P*. *dalatzensis* [44,45]. In fact, papillate subsidiary cells are common in *Pseudofrenelopsis* species, except for *P*. *gansuensis* and *P*. *intermedia* [50,51]. Papillae are common xeromorphic features of plants living under semi-arid climates and reduce the loss of water [55]. On the other hand, it was not possible to observe any guard cell, what precludes any inference on the orientation of the stomatal pits of the specimen described here.

Most studies on *Pseudofrenelopsis* did not focus on vascular structures, which may be related to the type of preservation of the analyzed species. Nevertheless, works like those of Sucerquia et al. [16], Axsmith [28], Hill et al. [54] and Zhou [51] showed some vascular features of fusainized specimens, and so does Alvin [53], who also presented data on the wood of silicified specimens. Such data are very important for paleoecological studies and can be quite informative regarding taxonomy [25].

As *P*. *parceramosa* and *P*. *intermedia* [28,51,53,54], LPU 312 PL presented tracheids arranged in longitudinal rows, but growth rings were not as well marked as those reported by the aforementioned studies. This fact is likely due to the developmental stage of the stem, which might have been young given that it was relatively thin (6 mm) and, hence, latewood cells were at the initial developmental stages. This can be observed in Fig 3C, where the cells closer to the cambial zone were more compact and had both reduced diameters and thicker walls. The transition from earlywood to latewood seemed to have been gradual, differing from the condition reported for *P*. *parceramosa* and *P*. *intermedia* [28,51,53,54] (Table 2). This feature might have been partially related to the availability of water resources and is not very appropriate for taxonomic purposes [56]. On the other hand, in the cortical region, secretory canals, here reported, were not observed in previous works.

**Table 2:** Comparison of stem anatomical features of *Pseudofrenelopsis* species.

Species	Growth ring	Tracheid pitting in radial walls	Cross-field type	Number of pits per cross field	Number of layers of cross-field pits	Secretory canals in the cortical region	References
*P*. *parceramosa*	Distinct, abrupt transition	Mostly uniseriate (rarely alternately biseriate)	Cupressoid	6–11	2–5	Absent	[28,53,54]
*P*. *capillata*	Not informed	Uniseriate, areolate	Cupressoid	More than 10	Not informed	Not informed	[16]
*P*. *intermedia*	Distinct, abrupt transition	Uniseriate, areolate	Cupressoid	4–6	2–3	Not informed	[51]
LPU 312 PL	Gradual transition, little distinguishable	Uniseriate, areolate	Intermediary between cupressoid and araucarioid	More than 10	5–6	Present	This paper

Due to difficulties faced by Axsmith [28] for studying thin fragments, he observed only structures of thicker and less fragile specimens. Even so, only few data were gathered. For instance, intervascular pits were seen from the inside of tracheids, hampering observations of the surface. In the present work, tracheids had their surface analyzed, including the preserved membranes, as also reported for *P*. *parceramosa* [54]. However, the central portion (torus) of the membranes of this taxon was thicker than in LPU 312 PL. The uniseriate intervascular pits are similar to those of *P*. *capillata*, *P*. *parceramosa* and *P*. *intermedia* [16,51,54]. After the classification of Phillipe and Bamford [26], the features seen in LPU 312 PL are typical of the abietinean radial pitting.

In cross-fields, *P*. *parceramosa*, *P*. *capillata* and *P*. *intermedia* had cupressoid pits with rounded, elliptical, or oblique openings [16,28,51,53,54]. Also, in *P*. *intermedia* there were only two to three rows of pits. On the other hand, in LPU 312 PL, individual pits had almost round openings, which are not typical of any category proposed by IAWA Committee [25], but approach the cupressoid type. However, their cross-field pitting type was comparable to the araucarioid one, in which there were ten or more pits per cross-field, distributed in four or five rows with tendency for crowding. The difficulty of attributing the condition of LPU 312 PL to any specific type may be related to the classification of IAWA Committee [25] based on extant species and/or due to the indistinctness between the araucarioid and cupressoid types after the fossilization [57].

LPU 312 PL shares many traits with other members of *Pseudofrenelopsis*, but it is characterized by a set of features that makes it well different from the aforementioned taxa, as shown in Tables 1 and 2. Thus, it is reasonable to state that the specimen in question is not *P*. *capillata*, which supports the inference of more than one *Pseudofrenelopsis* species in the Crato Formation of the Araripe Basin.

### Significance of *Brachyphyllum obesum* anatomical features

*Brachyphyllum* branches, despite being the most common plant megafossils in the Araripe Basin, are understudied regarding their anatomy. Rare exceptions, like Kunzmann et al. [21] and Sucerquia [22,23], covered this subject. So far, only two species based on leafy branches have been recognized from that basin, *B*. *obesum*, by far the most abundant one, and *B*. *castilhoi*, rarely found [15].

Some features could not be observed due to the poor preservation of some specimens, so they will not be discussed here. Among the leaf anatomical traits, the shape and arrangement of stomata are noteworthy. A great number of *Brachyphyllum* species resembles *B*. *obesum* from the Araripe Basin in some aspects. *B*. *obesum* specimens from the Tetori Group, Japan, described by Yabe and Kubota [20], had their leaf surface ornamented by longitudinal striations that converged towards the apex. These striations, also present in the Araripe specimens and many other species (*e*.*g*., *B*. *lorchi*, *B*. *ningshiaense*, *B*. *obtusum*, *B*. *squamosum*, *B*. *negevensis*, *B*. *expansum*, and *B*. *scalbiensis*), are more precisely stomatal rows [12,13,58–61]. In contrast, many other species presented stomata arranged in a random or varied fashion, like *B*. *patens*, *B*. *tigrense*, *B*. *elegans*, *B*. *pulcher*, and *B*. *castatum* [6,58,62–64], or their stomatal rows are not well defined, as in *B*. *mamillare* and *B*. *crucis* [13]. A large number of species with irregularly distributed stomata were attributed to Cheirolepidiaceae, whereas most species with longitudinal stomatal rows were allocated within Araucariaceae [6,11,65] (Table 3).

**Table 3:** Comparison of anatomical features of *Brachyphyllum obesum*, Araucariaceae, and Cheirolepidiaceae.

Taxon	Stomatal arrangement	Tracheid pitting in radial walls	Cross-field type	Secretory channels in xylem	Refereces
Cheirolepidiaceae	Usually random or rarely arranged in rows	Usually uniseriate	Usually cupressoid	Not informed or absent	[6,16,28,46,51,54,62,64]
Araucariaceae	Usually in continuous longitudinal rows or rarely random	Often biseriate, alternate, and/or more rarely uniseriate	Araucarioid	Resin plugs are common	[11,13,25,65–68]
*B*. *obesum*	In continuous longitudinal rows	Biseriate, alternate, or uniseriate	Araucarioid	Resin plugs eventually found	This paper,[15,21,23]

Most *Brachyphyllum* species from other regions had completely sunken stomatal apparatus [6,58,59,61]. Particularly, in *B*. *obesum* specimens here described, the anticlinal walls of the subsidiary cells were prominent and only the guard cells, which were not preserved, could have been sunken in relation to other epidermal cells, as in *B*. *mamillare* and *B*. *stemonium*, schematically represented by Kendal [13]. This feature is quite common in arid environments, because it avoids a greater loss of water by means of evapotranspiration [49,69]. Despite the non-observation of stomatal pits, the general shape of the stomatal apparatus suggest perpendicular openings, which is in accordance with the specimens analyzed by Kunzmann et al. [21].

As mentioned for *Pseudofrenelopsis*, works on wood structures of *Brachyphyllum* are rare [6,11], which might be also related to difficulties for processing brittle specimens. The study of Kunzmann et al. [21] reported some features of the *B*. *obesum* xylem, but they did not scrutinize them or present more detailed images. On the other hand, the new specimens here described partially fill in the gap in the knowledge of xylem features of *B*. *obesum* from the Crato Formation. The described samples show a xylem composed by oval to round tracheids with uniform diameters and few intercellular spaces, being similar to many gymnosperm species, including *B*. *macrocarpum*, *Araucarioxylon chapmanae*, and *Araucariopitys antarcticus* [11,66]. Furthermore, tracheid walls were thin and the lumina were ample, as in *A*. *chapmanae* and *A*. *antarcticus* [66]. However, there is no evidence of different growth patterns, differing from the latter two species but similar to the *B*. *obesum* specimens figured by Sucerquia [23]. Particularly, the absence of growth rings might have been related to either the age of the stem, without having gone completely through a seasonal cycle, or a period of water stability [70–72].

Pits observed in the studied samples were areolate, as those reported in *B*. *obesum* [23], *B*. *macrocarpum* [11], and *B*. *patens* [6]. In addition, the specimens figured by Sucerquia [23] presented biseriate and alternate pits, a common feature of members of Araucariaceae [25,66]. Nevertheless, here we reported tracheids bearing uniseriate pits with angular outlines in *B*. *obesum*, which were also observed in *A*. *antarcticus* [66].

Resin plugs, eventually noticed, are common tracheid features of members of Araucariaceae [25], whereas wall thickenings are rarer in the fossil record. With respect to the *B*. *obesum* specimens analyzed here, the observed thickenings resembled those of *B*. *patens* [6]. On the other hand, each cross-field had six or more pits distributed in three or four rows and with a tendency for crowding and, hence, could be classified as of the araucarioid type [25]. However, this condition differs from the average of eleven pits per field reported by Sucerquia [23]. The significance of this dissimilarity between different *B*. *obesum* specimens is something to be further investigated.

The taxonomic placement of *B*. *obesum* specimens from the Araripe Basin has been controversial. They are sometimes allocated in Cheirolepidiaceae, due to morphological resemblances with non-frenelopsid members of this family and the abundance of *Classopollis*-type pollen [22,73]. On the other hand, they are also included in Araucariaceae based on morphological and epidermal features [21,23,73]. The present study on *B*. *obesum* from the Crato Formation of the Araripe Basin supports in general its allocation within Araucariaceae, following Kunzmann et al. [21] and Sucerquia [23], based on both epidermal and wood features that are exclusive or more common in this family, like: 1) stomata arranged in longitudinal rows; 2) alternate tracheid pits; 3) presence of resin plugs; 4) araucarioid cross-field pits.

### Paleoecological significance

All specimens studied in this contribution show adaptations conditioned by aridity and/or salinity [16,21]. It should be noted that the effects of these factors may be difficult to be distinguished from each other, because the adaptive responses for the water stress in both conditions can be the same [73,74]. In *Brachyphyllum obesum*, the small, imbricate, and appressed leaves were likely responses to the intense solar radiation and water scarcity, reducing the surface for gas exchanges and, hence, the water loss during the evapotranspiration [73,75]. Although not preserved, *Pseudofrenelopsis* sp. (LPU 312 PL) leaves seemed to have been less numerous than those of *B*. *obesum* as indicated by their insertion points. This fact may suggest that adaptive responses to the water stress of the former conifer were even more extreme than in the latter.

Vascular features might have had also paleoecological implications. For instance, in *Pseudofrenelopsis* sp., the thick tracheid walls could have prevented the collapse of the conducting cells, indicating great tolerance to the water scarcity [76–79]. Also, the thick membranes between the pits of *Pseudofrenelopsis* sp. could also avoid the embolism of the conducting system, being another indicator of hydraulic safety [76,77,80]. These membranes were not yet reported for *P*. *capillata*, but they are even thicker in *P*. *parceramosa*, from the Lower Cretaceous Speeton Clay Formation, especially at their central portion (torus) [54]. This difference regarding the thickness of the membrane may imply that *P*. *parceramosa* inhabited areas under more intense water stress than the specimen from the Araripe Basin here discussed.

On the other hand, *B*. *obesum* tracheids presented less thick walls and greater lumina than those of LPU 312 PL, which could be related to different paleoenvironmental settings, including the distance from their specific habitats to the depositional environment. However, the evidence at hand is scanty, especially regarding the unknown stratigraphic provenance of the specimens investigated here within the Crato Formation. Thus, other approaches are mandatory in order to test that hypothesis, including comparisons with other taxa, like *P*. *capillata*.

## Final remarks

This study sheds new lights on the anatomy of stems and leaves of two conifers from the upper Aptian Crato Formation of the Araripe Basin, *Pseudofrenelopsis* sp. and *Brachyphyllum obesum*. For instance, it was described for the first time a *Pseudofrenelopsis* specimen from the aforementioned deposit without papillate epidermal cells in the middle region of the internodes, but with tracheids presenting the abietinean radial pitting and round cross-field pits arranged in a pattern intermediary between the cupressoid and the araucarioid types. On the other hand, this analysis identified in *B*. *obesum* stomatal rows, tracheids with biseriate and alternate pits, resin plugs, and araucarioid cross-field pits. Besides their physiological and ecological implications, the new data have also important taxonomic implications for both genera. In the case of *Pseudofrenelopsis*, it is likely that at least two different species were present in the Crato Formation taphoflora, whilst the aforementioned features of *B*. *obesum* further support its previous placement among araucariaceans. However, these inferences can be seen as hypotheses for future investigations and any contribution in this regard is welcomed.
